# *Oprm1* A112G, a single nucleotide polymorphism, alters expression of stress-responsive genes in multiple brain regions in male and female mice

**DOI:** 10.1007/s00213-018-4965-x

**Published:** 2018-07-19

**Authors:** Devon Collins, Matthew Randesi, Joel Correa da Rosa, Yong Zhang, Mary Jeanne Kreek

**Affiliations:** 1 The Laboratory of the Biology of Addictive Diseases, The Rockefeller University, 1230 York Avenue, New York, NY 10065 USA; 2 Laboratory of Investigative Dermatology, The Rockefeller University, 1230 York Avenue, New York, NY 10065 USA

**Keywords:** Oprm1 A112G, Mu-opioid receptor, Physiological and behavioral responses, Gene expression

## Abstract

**Background:**

OPRM1 A118G, a functional human mu-opioid receptor (MOR) polymorphism, is associated with drug dependence and altered stress responsivity in humans as well as altered MOR signaling. MOR signaling can regulate many cellular processes, including gene expression, and many of the long-term, stable effects of drugs and stress may stem from changes in gene expression in diverse brain regions. A mouse model bearing an equivalent polymorphism (Oprm1 A112G) was previously generated and studied. Mice homozygous for the G112 allele show differences in opioid- and stress-related phenotypes.

**Approach:**

The current study examines the expression of 24 genes related to drug and stress responsivity in the caudoputamen, nucleus accumbens, hypothalamus, hippocampus, and amygdala of drug-naïve, stress-minimized, male and female mice homozygous for either the G112 variant allele or the wild-type A112 allele.

**Results:**

We detected nominal genotype-dependent changes in gene expression of multiple genes. We also detected nominal sex-dependent as well as sex-by-genotype interaction effects on gene expression. Of these, four genotype-dependent differences survived correction for multiple testing: *Avp* and *Gal* in the hypothalamus and *Oprl1* and *Cnr1* in the hippocampus.

**Conclusions:**

Changes in the regulation of these genes by mu-opioid receptors encoded by the G112 allele may be involved in some of the behavioral and molecular consequences of this polymorphism observed in mice.

## Introduction

Mu opioid receptors are involved in diverse neurobiological functions including drug reward, analgesia, and stress responsivity. The mu-opioid receptor (MOR) is the primary site of action of many of the endogenous opioids, including β-endorphin and met-enkephalin (Akil et al. 1984; Champion et al. 1997; Hughes et al. 1975) and many clinically important opioid therapeutics. Physiological and behavioral responses to opioids can vary from individual to individual (Smith 2008), and the heritability of complex, drug-induced phenotypes suggests a critical role of genetic influences (Kreek et al. 2005, 2012; Al-Hasani and Bruchas 2011).

A common single-nucleotide polymorphism (SNP) in the human mu-opioid receptor gene (*OPRM1*), A118G, is associated with drug dependence in humans (Bart et al. 2004, 2006; Bond et al. 1998; Nishizawa et al. 2006), although some studies have found no association (Bergen et al. 1997; Coller et al. 2009), and others have suggested that it may be associated with drug dependence in some populations but protective in others (Schwantes-An et al. 2016). A118G is the most common coding region variant in the *OPRM1* gene. Its overall allelic frequency is estimated to be 22%, although its distribution shows significant variation by ancestry: 1% in Africans, 20% in indigenous Americans, 39% in East Asians, 42% in South Asians, and 16% in Europeans (Aken et al. 2016; LaForge et al. 2000).

The G118 allele is associated with greater daily drug intake in heroin users (Shi et al. 2002), greater automatic approach tendencies for alcohol and appetitive cues (Wiers et al. 2009), and increased neuronal activity in response to alcohol cues in cortical and striatal regions as shown by BOLD (blood oxygenation level dependent) imaging (Filbey et al. 2008). The G118 allele also significantly alters stress responsivity and HPA axis regulation in healthy humans. G118 carriers show genotype-dependent differences in response to endocrine challenge by metyrapone (Ducat et al. 2013) as well as reductions in cortisol response to acute psychological stressors and MOR blockade by naltrexone or naloxone (Chong et al. 2006; Hernandez-Avila et al. 2003; Lovallo et al. 2015; Wand et al. 2002).

*OPRM1* A118G has been demonstrated to produce a number of biochemical and molecular alterations (Kroslak et al. 2007). The G118 allele is associated with reduced dynorphin and enkephalin gene expression and increased peptide levels in heroin users (Drakenberg et al. 2006). In vitro studies in AV12 cells expressing the G118 MOR variant revealed a three-fold increase in β-endorphin binding affinity as well as a three-fold increase in β-endorphin potency as measured by activation of G protein-coupled inward rectifying potassium channels (Bond et al. 1998).

Mice bearing an equivalent SNP, A112G, in the mouse MOR gene (*Oprm1*) were generated, characterized, and studied (Mague et al. 2009). The *Oprm1* A112G mouse exhibits genotype-dependent molecular alterations, including reductions in MOR mRNA and protein expression (Huang et al. 2012; Mague et al. 2009; Wang et al. 2014) as well as behavioral alterations, including reduced morphine (Huang et al. 2012; Mague et al. 2009; Wang et al. 2014) and buprenorphine (Browne et al. 2017) analgesia, and increased opioid self-administration (Zhang et al. 2015).

MOR activation leads to a series of downstream cellular effects (Al-Hasani and Bruchas 2011), which may include changes in gene expression. Many of the long-term changes in brain and behavior that characterize addiction are likely underpinned by changes in gene expression (Nestler 2004a; Nestler and Aghajanian 1997). As the A118G variant alters MOR function, it may cause changes in MOR-modulated gene expression that contribute to the behavioral, molecular, and neurochemical alterations observed in humans and rodents bearing the G allele (Kreek et al. 2005; Mura et al. 2013).

In the current study, we have profiled the expression of an array genes that (1) are associated with mu opioid dependence in humans (Bart et al. 2004; Levran et al. 2008) or (2) have been demonstrated to be related to responses to stress or drugs of abuse in rodents (Bale et al. 2000; Caputi et al. 2014; Deroche-Gamonet et al. 2003; Edwards et al. 2012; McClung et al. 2005; Spangler et al. 1993; Uhl et al. 1988; Valenza et al. 2016; Zhou et al. 2001). Animal and human studies show that addiction involves diverse circuits widely distributed across the brain including striatal, limbic, and hypothalamic regions (Koob and Volkow 2010). Here, we measure the baseline mRNA expression of these genes in the caudoputamen, nucleus accumbens, hypothalamus, hippocampus, and amygdala in minimally handled, drug-naïve, male, and female mice homozygous for either the A112 (AA) or G112 (GG) allele. This is the first measurement of the concurrent expression of multiple genes in multiple brain regions in this mouse model, and, to our knowledge, one of very few studies examining the effects of a single human SNP—successfully translated into a rodent model—on the expression of multiple genes in multiple brain regions.

## Materials and methods

### Animals

Heterozygous A112G mice (Mague et al. 2009) were mated to obtain homozygous (112AA or 112GG; AA or GG) offspring. Heterozygous mice (112AG) were also produced, but only homozygous mice of both sexes are examined in the present study. Mice were genotyped by PCR using genomic DNA obtained by tail biopsy (forward primer, 5′-GCTCCA TCTTGGATCCCCTTT-3′; reverse primer, 5′-GAGCTACCCAGCAATTCCAGA-3′). Ten- to twelve-week-old mice were housed in groups of four to five with free access to food and water in a light- (12:12 h light/dark cycle, lights on at 0900, lights off at 2100) and temperature- (25 °C) controlled room. Animal care and experimental procedures were conducted according to the *Guide for the Care and Use of Laboratory Animals* (National Research Council (US) Committee for the Update of the Guide for the Care and Use of Laboratory Animals, 2011). The experimental protocols used were approved by the Institutional Animal Care and Use Committee of The Rockefeller University.

### RNA extraction and cDNA synthesis

Mice were sacrificed by rapid decapitation between 2 and 3 h following lights on (1100–1200). Whole brains were removed and the caudoputamen, nucleus accumbens, hypothalamus, hippocampus, and amygdala from each were dissected. Tissues were homogenized in QIAzol (Qiagen, Valencia, CA). Total RNA was isolated from homogenates using miRNeasy kits (Qiagen, Valencia, CA). RNA quality and quantity for each sample were determined using an Agilent 2100 Bioanalyzer (Agilent Technologies, Santa Clara, CA). Genomic DNA was removed and cDNA was synthesized from 500 ng of total RNA using RT^2^ HT First Strand kits (Qiagen, Valencia, CA).

### Custom RT^2^ Profiler™ PCR Array

Our custom RT^2^ Profiler™ PCR Array (plate build CAPM13405E, Qiagen) used for the present study measures the expression of 24 genes related to drug and stress responsivity (for complete list, see Table 1). Real-time PCR was performed by the SYBR Green detection method. Individual reactions had a total volume of 10 μL and comprised cDNA diluted in 2× SuperArray RT^2^ Real-Time™ SYBR Green PCR Master Mix (Qiagen) and ultrapure water.

**Table 1:** Genes comprising the custom RT2 Profiler™ PCR Array

Group	Gene symbol	Gene name
Opioid	*Oprm1*	Mu opioid receptor
	*Pomc*	Proopiomelanocortin
	*Oprk1*	Kappa opioid receptor
	*Pdyn*	Preprodynorphin
	*Oprd1*	Delta opioid receptor
	*Penk*	Preproenkephalin
	*Oprl1*	Opioid-related nociceptin receptor
	*Pnoc*	Prepronociceptin
Stress	*Avpr1b*	Arginine vasopressin receptor 1B
	*Avp*	Arginine vasopressin
	*Crhr1*	Corticotropin releasing hormone receptor 1
	*Crh*	Corticotropin releasing hormone
	*Nr3c1*	Glucocorticoid receptor
	*Nr3c2*	Mineralocorticoid receptor
	*Mc2r*	Melanocortin receptor 2
	*Cnr1*	Cannabinoid receptor 1
	*Cnr2*	Cannabinoid receptor 2
	*Faah*	Fatty acid amide hydroxylase
	*Fkbp5*	FK506 binding protein 5
	*Galr1*	Galanin receptor
	*Gal*	Galanin
	*Oxtr*	Oxytocin receptor
	*Oxt*	Oxytocin
	*Csnk1e*	Casein kinase 1 epsilon
Reference genes	*Gapdh*	Glyceraldehyde-3-phosphate dehydrogenase
	*Hsp90ab1*	Heat shock protein 90 alpha (cytosolic), class B member 1
	*Tbp*	TATA box binding protein
	*Gusb*	Glucuronidase, beta
	*Ppia*	Peptidylprolyl isomerase A (cyclophilin A)

The real-time PCR reactions were carried out in an ABI Prism 7900 HT Sequence Detection System using the following program: 10 min at 95 **°**C (15 s at 95 **°**C and 1 min at 60 **°**C) × 40 cycles, 15 s at 95 **°**C, 15 s at 60 **°**C, and 15 s at 95 **°**C. The ABI Prism 7900 Sequence Detection System was used to calculate the Ct value for each well. The profiler array includes five reference genes (glyceraldehyde 3-phosphate dehydrogenase, *Gapdh*; β-glucuronidase, *Gusb*; heat shock protein 90 alpha (cytosolic), class B member 1, *Hsp90ab1*; peptidylprolyl isomerase A, *Ppia*; TATA binding protein, *Tbp*). Of these five reference genes, *Gusb* was stably expressed across groups in caudoputamen and *Tbp* was stably expressed across groups in nucleus accumbens, hypothalamus, hippocampus, and amygdala. Ct values were normalized by calculating the difference between the target gene and the most stable reference gene according to brain region.

### Statistical analysis

The statistical analysis focused on effects of sex and genotype on the expression of genes of interest in each brain region. A sample of *n* = 20 mice (representing approximately 9 litters) was allocated in four different groups (male AA, male GG, female AA, female GG; *N* = 5/group) according to a 2 × 2 factorial design. Normalized RT-PCR gene expressions were analyzed by two-way ANOVA with main effects of sex and genotype as well as their interaction. Whenever the interaction was found to be significant, post hoc nested comparisons were carried out (e.g., difference between females and males within genotype or difference between genotypes within sex). If no interaction was found to be significant, significant differences on the main effects were reported. Genes showing significant effects with nominal *p* values are reported in Tables 2, 3, and 4. The Benjamini-Hochberg method (false discovery rate; 5%) was used to identify differences in gene expression remaining significant after correction for multiple hypothesis testing (see Tables 2, 3, and 4).

**Table 2:** Genes differing by *Oprm1* genotype

Region	Gene	Direction of change	Ratio^a^	Nominal *p* val.	Corrected p val.^b^
Caudoputamen					
	*Oprm1*	↓	0.88	0.02	0.26
	*Pdyn*	↓	0.82	0.01	0.26
N. accumbens					
	*Gal*	↓	0.46	0.03	0.60
Hypothalamus					
	*Oprk1*	↓	0.85	0.04	0.16
	*Pdyn*	↓	0.82	0.03	0.16
	*Penk*	↓	0.80	0.03	0.16
	*Avp*	↓	0.60	0.0002	0.004*
	*Gal*	↓	0.76	0.002	0.03*
	*Oxt*	↓	0.68	0.008	0.07
Hippocampus					
	*Oprl1*	↓	0.72	0.002	0.03*
	*Cnr1*	↓	0.70	0.001	0.03*
	*Fkbp5*	↓	0.82	0.04	0.23
	*Oxtr*	↓	0.66	0.049	0.23
Amygdala	–	–	–	–	–

^a^GG vs AA (prototype)

^b^False discovery rate (5%)

*Remains significant after adjustment for multiple testing (FDR = 5%)

**Table 3:** Genes differing by sex

Region	Gene	Direction of change^a^	Ratio^b^	Nominal *p* val.	Corrected p val.^c^
Caudoputamen					
	*Pnoc*	↑	1.38	0.04	0.23
	*Avp*	↑	3.39	0.003	0.08
	*Crh*	↑	1.30	0.02	0.20
	*Gal*	↑	1.96	0.03	0.20
	*Oxtr*	↑	1.47	0.047	0.23
N. Accumbens	–	–	–	–	–
Hypothalamus					
	*Gal*	↑	1.21	0.03	0.62
Hippocampus	*Oxtr*	↑	1.60	0.03	0.50
Amygdala	–	–	–	–	–

^a^Female vs male

^b^Female/male

^c^False discovery rate (5%)

**Table 4:** Genes showing a genotype-by-sex interaction

Region	Gene	Effect (contrasts nested)	Direction of change	Ratio	Nominal *p* val.	Corrected *p* val.^a^
Caudoputamen	*Pdyn*	*Overall*			0.03	0.14
		within males (GG vs AA)	↓	0.68	0.002	
	*Cnr1*	*Overall*			0.009	0.56
		within males (GG vs AA)	↓	0.72	0.008	
		within AA (F vs M)	↓	0.75	0.01	
	*Nr3c2*	*Overall*			0.03	0.54
		within males (GG vs AA)	↓	0.83	0.06	
N. accumbens	*Cnr2*	*Overall*			0.02	0.54
		within males (GG vs AA)	↓	0.35	0.004	
Hypothalamus	*Oprk1*	*Overall*			0.006	0.14
		within females (GG vs AA)	↓	0.68	0.00	
		within AA (F vs M)	↑	1.27	0.03	
	*Pdyn*	*Overall*			0.01	0.14
		within females (GG vs AA)	↓	0.65	0.002	
		within AA (F vs M)	↑	1.46	0.005	
Hippocampus	–	–	–	–	–	–
Amygdala	*Gal*	*Overall*			0.01	0.43
		within males (GG vs AA)	↑	3.19	0.01	
		within AA (F vs M)	↑	2.58	0.04	

^a^False discovery rate (5%)

## Results

### *Oprm1* A112G mice

We have observed no differences in size, health, or mortality between AA and GG mice of either sex (data not shown). GG mouse birth rates are slightly lower than the expected Mendelian rates as has been previously described (Mague et al. 2009); however, mice carrying the 112G allele are fertile and produce litters of normal size. We used both male and female homozygous mice (AA or GG) in the current study.

Our primary aim was to identify genes that were differentially expressed in GG versus AA mice in each brain region. We also sought to identify genes included in our array that are differentially expressed in females and males, as the expression of some of the target genes included in our custom array might differ by sex, and because some studies have found sex-dependent differences in behavioral and molecular measures in *Oprm1* A112G mice (Mague et al. 2009; Wang et al. 2014). Finally, we examined the interaction of sex and genotype. Findings are summarized in Tables 2, 3, and 4.

## Basal gene expression in *Oprm1* A112G mice

Our initial analyses detected genotype- and sex-dependent differences in the expression of multiple genes in the brain regions examined. Many of these findings did not survive correction for multiplicity of testing; however, we report them here for exploratory purposes. Differences in expression remaining significant after correction for multiple testing (FDR; 5%) are reported below.

### Effect of genotype on basal gene expression

We detected nominally significant genotype-dependent differences in expression of two genes in caudoputamen, one gene in nucleus accumbens, six genes in hypothalamus, and four genes in hippocampus. In amygdala, we did not observe any significant differences in gene expression between AA and GG mice. Following correction for multiple testing, two genes differing by genotype in hypothalamus, *Avp* and *Gal*, and two genes differing by genotype in hippocampus, *Cnr1* and *Opr1*, remained significantly different between AA and GG mice (Table 2).

### Effect of sex on basal gene expression

We detected nominally significant sex-dependent differences in the expression of five genes in caudoputamen, one gene in hypothalamus, and one gene in hippocampus. We did not observe any significant main effects of sex on the expression of any genes in nucleus accumbens or amygdala. None of these sex-dependent differences in gene expression survived correction for multiple testing; however, the nominally significant findings are reported in Table 3.

### Interaction of genotype and sex

We observed nominally significant interaction effects of genotype and sex on the expression of three genes in caudoputamen, one gene in nucleus accumbens, two genes in hypothalamus, and one gene in amygdala. We did not observe any interaction effects in gene expression in hippocampus. None of these nominally significant interactions survived correction for multiplicity of testing; however, we report them in gene (Table 4) in amygdala.

## Discussion

Alterations in gene expression likely contribute to long-term, stable changes and variability in drug- and stress-mediated behavior (Gray et al. 2014; Kreek et al. 2005, 2012; Nestler 2004b; Nestler and Aghajanian 1997); these differences in gene expression may then contribute to alterations in drug- and stress-related behaviors in *Oprm1* A112G mice. In the current study, we examined the basal expression of 24 genes that have been demonstrated to be regulated by exposure to major drugs of abuse or stress (by our own group or others). Although we found nominally significant genotype- and sex-dependent differences in the basal expression of a number of these genes in multiple regions, only genotype-dependent differences in the expression of *Avp* and *Gal* in the hypothalamus, as well as *Oprl1* and *Cnr1* in the hippocampus survived corrections for multiplicity of testing (Tables 2, 3, and 4). This is likely due to the relatively small sample size used in this study. Future studies with greater statistical power may reveal other genotype-dependent differences as well as sex-dependent differences in gene expression in male and female AA and GG mice.

The hypothalamus plays a large role in feeding, stress, and homeostatic behavior, but it may also modulate reward-related behaviors (DiLeone et al. 2003; Le Merrer et al. 2009). Interestingly, injection of an enkephalin analog into the hypothalamus can produce conditioned place preference (Agmo and Gómez 1991), suggesting that mu opioid receptor signaling in the hypothalamus is involved in conditioned reward. We detected genotype-dependent differences in hypothalamic expression of the genes encoding the non-opioid neuropeptides arginine vasopressin and galanin. *Avp* and *Gal* expressions were reduced in GG mice. These neuropeptides are involved in diverse functions including stress responsivity, motivational effects of opioids and psychostimulants, processing of appetitive stimuli, and emotional regulation (Bali et al. 2015; Bisagno and Cadet 2014; Zhou et al. 2008).

AVP in the central nervous system is involved in stress responsivity, drug dependence, and emotional behavior. In the neurohypophyseal system, AVP acts as an adrenocorticotropic hormone (ACTH) secretagogue Aguilera et al. 1994; Griebel et al. 2002; Zhou et al. 2008) and, along with corticotropin releasing hormone, AVP is involved in regulating hypothalamic-pituitary-adrenal (HPA) axis function. There is evidence that AVP also participates in the effects of opioids on HPA activity. Precipitated morphine withdrawal can lead to alterations in *Avp* expression (Nunez et al. 2007) and naloxone can reduce hypothalamic AVP mRNA levels, suggesting regulation by endogenous opioids (Zhou et al. 2005). Hypothalamic galanin is implicated in stress resiliency (Juhasz et al. 2014; Wrenn and Holmes 2006) and altered galanin expression in this region has been associated with multiple pathological states, including depression and alcoholism (Davidson et al. 2011). Both chronic morphine and morphine withdrawal have been demonstrated to alter galanin gene expression in multiple brain regions, implicating MORs in regulation of *Gal* expression (McClung et al. 2005).

The hippocampus is critically involved in learning and memory, but may also be involved in motivated behavior and drug reinforcement (Ito et al. 2008; Koob and Volkow 2010; Le Merrer et al. 2009; Sharifzadeh et al. 2006; Tracy et al. 2001). We found reduced hippocampal expression of *Oprl1*, *Cnr1* in GG mice. There is evidence that these are involved in hippocampal function. Hippocampal infusions of nociceptin can impair spatial learning (Sandin et al. 1997). Intracerebroventricular nociceptin can abolish morphine conditioned place preference (Ciccocioppo et al. 2000), which involves the hippocampus (Huston et al. 2013). Cannabinoid receptor signaling, including CB_1_ signaling, can regulate hippocampal function, presumably by regulating neurotransmitter release and synaptic responses (Davies et al. 2002).

The hippocampus plays a role in feedback inhibition of the HPA axis (Jacobson and Sapolsky 1991). Healthy human G118 carriers have altered HPA axis function Chong et al. 2006; Ducat et al. 2013; Hernandez-Avila et al. 2003; Lovallo et al. 2015; Wand et al. 2002) and GG mice show resilience to social stress (Briand et al. 2015), which may, in part, reflect altered hippocampal regulation of the HPA axis.

Interestingly, a study of hippocampal function in A112G mice has shown that wild-type mice have increased excitatory hippocampal activity in response to morphine. This effect is attenuated in GG mice and may be due to a MOR loss of function in hippocampus (Mague et al. 2015). These changes may result from a reduction in direct effects of MORs on hippocampal neurons as well as indirect effects through nociceptin or cannabinoid signaling.

Sex differences in the expression of drug of abuse- and stress-responsive genes in male and female mice are not routinely studied. Genotype-by-sex effects on behavior have been observed in A112G mice. For example, Mague and colleagues found that female GG mice fail to develop a significant morphine conditioned place preference, while AA males, AA females, and GG males did develop a significant morphine conditioned place preference. In the same study, GG females also showed reduced naloxone-precipitated morphine withdrawal compared to AA females (Mague et al. 2009). Further, GG mice also show brain-region and sex-specific alterations in DAMGO-stimulated GTPγS binding (Wang et al. 2014). We observed several nominally significant sex-dependent differences in gene expression and genotype-by-sex interactions; however, none of these findings survived correction for multiple testing. Interestingly, for all of these nominally significant sex-dependent differences in gene expression, female mice showed greater expression levels compared to males regardless of genotype (Table 3). It is unclear why this may be the case; however, it has been demonstrated that mu opioid effects and mu opioid receptor signaling can vary between male and female rodents (Craft 2008). Both gene expression (Beato 1989) and opioid receptor availability can be modulated by hormonal status in at least some brain regions (Zubieta et al. 1999). To date, there have been no studies of the effects of hormonal status in *Oprm1* A112G mice. Studies involving the manipulation of gonadal steroids in these mice will be required to investigate their involvement in the sex-dependent differences observed in A112G mice.

### Potential mechanisms of regulation of gene expression by MORs

MORs are poised to regulate a number of transcriptional pathways by regulating the activity of several downstream effectors, including adenylate cyclase, ion channels, and, of note, multiple classes of kinases (Ho et al. 2009). Many of the signaling pathways that MORs modulate ultimately act to regulate the activity of well-characterized transcriptional regulators such as NF-κB, CREB, and Fos proteins (Al-Hasani and Bruchas 2011; Deb et al. 2010; Goldsmith et al. 2011; Ho et al. 2009), although the ultimate transcriptional consequences depend upon cellular context. For example, acute morphine can increase NF-kB activity, subsequently altering the expression of opioid receptors and peptides, while chronic morphine can lead to a reduction in NF-kB activity (Chen et al. 2006). In mice, CREB signaling increases in the ventral tegmental area in response to nicotine reward-associated environments and this increase requires MORs (Walters et al. 2005). In A112G mice, compared to wild-type AA homozygotes, GG mice show increased c-Fos immunoreactivity in multiple brain regions in following social stress (Briand et al. 2015). Interestingly, in the current study, for all genes showing a main effect of genotype, the G112 allele led to a reduction in gene expression. This suggests reduced MOR-dependent positive transcriptional regulation by MORs encoded by the variant G112 allele compared to the prototype A112 allele.

## Conclusion

We have demonstrated that the basal expression of genes encoding key components of drug of abuse- and stress-responsive systems are altered in male and female *Oprm1* A112G mice. These data provide evidence that the effects of the A118G SNP likely involve differential regulation of multiple, interacting neuropeptide systems. Future investigation of the downstream signaling consequences of MORs in A112G mice will be valuable to understanding the behavioral and molecular changes caused by the G allele in rodent models and in humans.
